# 2563 National dissemination of the accrual to clinical trials (ACT) network across the Clinical and Translational Science Award (CTSA) Consortium*

**DOI:** 10.1017/cts.2018.172

**Published:** 2018-11-21

**Authors:** Elaine H. Morrato, Lindsay Lennox

**Affiliations:** University of Colorado at Denver

## Abstract

OBJECTIVES/SPECIFIC AIMS: The ACT Network was developed by 46 members of the CTSA Program hubs in collaboration with NCATS to help investigators explore and validate feasibility of clinical studies in real-time using linked electronic health record data for cohort discovery. ACT is being disseminated nationally across the CTSA consortium. METHODS/STUDY POPULATION: Diffusion of Innovation Theory and Lean Start-Up principles inform dissemination strategies. Core materials were developed nationally and are being tailored to meet local CTSA dissemination norms. An advisory board, with expertise in communications, journalism, customer channel management, pharmaceutical commercialization and health IT entrepreneurship, is providing strategic advice to develop and refine dissemination strategies. Evaluation of dissemination methods will include network usage and web analytics for the ACT Network’s interactive digital content and log-in portal, and surveys-interviews of ACT users using the RE-AIM implementation framework. RESULTS/ANTICIPATED RESULTS: Formative research identified ACT’s primary value proposition for clinical researchers: “Explore patient populations in depth, in real time, from your desktop;” “Confirm study feasibility by iteratively testing and refining inclusion and exclusion criteria;” “Demonstrate feasibility in funding proposals and IRB submissions;” and “Identify collaborating sites for multi-site studies by searching for patients across the CTSA network.” Early dissemination metrics, including number-type of registered users, queries performed, and web analytics, will be presented. DISCUSSION/SIGNIFICANCE OF IMPACT: Researchers nationwide face common barriers in accruing enough participants for clinical trials. The inability to identify the right number and type of people to participate often makes clinical trials slow and costly. Better cohort discovery at the protocol development phase is a necessary requirement. By end of 2018, the ACT Network will reach 60% of the CTSA consortium providing a new tool for investigators to improve the design and execution of clinical trials.

